# Cases of Yolk sac tumor associated with gynecological malignant tumor

**DOI:** 10.1186/s12905-023-02495-w

**Published:** 2023-06-30

**Authors:** Shengchao Wang, Kelie Chen, Qin Chen, Shuai Huang, Weiguo Lu

**Affiliations:** Department of Gynecological Oncology, Women’s Hospital, Zhejiang University School of Medicine, Hangzhou, 310006 Zhejiang China

**Keywords:** Yolk sac tumour (YST), Malignant gynaecological tumours, Treatment, Chemotherapy, Prognosis

## Abstract

**Background:**

Yolk sac tumour (YST) is the second most common ovarian germ cell tumour and usually presents in children and young women. However, tumours rarely occur as malignant gynaecological tumours with YST components.

**Case presentation:**

We present one case of endometrioid carcinoma and clear cell carcinoma with YST components and two other cases of YSTs associated with high-grade serous carcinoma of the ovary in females. After surgery and adjuvant chemotherapy, the patient with endometrioid carcinoma had progressive disease and died 20 months later, and the other two were still alive at the last follow-up.

**Conclusions:**

To our knowledge, these mixed neoplasm associations are unusual, and these cases illustrate the diagnosis and prognosis of YST associated with malignant gynaecological tumours, emphasizing early recognition and aggressive treatment.

## Background

Yolk sac tumour (YST) is the second most common ovarian germ cell tumour and usually occurs in infants and adolescents [1, 2]. These tumours are typically found in the gonads and areas with a primitive extraembryonic morphology, but YSTs involving the endometrium and combined with epithelial ovarian carcinoma are extremely rare [3, 4]. There are no guidelines for the treatment of malignant gynaecological tumours with YST components. At present, surgery and chemotherapy are the major therapies and appear to be associated with a poor outcome [5, 6]. Herein, we present three cases of YST involving the endometrium and coexisting with malignant ovarian tumours and report on the diagnosis and prognosis of these patients after systemic therapies (Table 1).

**Table 1:** Clinical Features of Cases of Yolk sac tumor associated with gynecological malignant tumor

Patient	Age	Stage	AFP (ng/mL)	Pathology	surgery	chemotherapy	Follow-Up
Case 1	36	IVb	>1000	EC + CCC + YST (G2)	TAH + BSO + PLN + PALN + omentectomy + tumor debulking(complete cytoreduction)	TP (3 cycles)	Progression during chemotherapy Death:20 mo
Case 2	55	IIa	1190	YST + HGSC (Ovarian)	TAH + BSO + PLN + PALN + omentectomy After recurrence: tumor debulking(complete cytoreduction)	TP (6 cycles) After recurrence: TP (6 cycles)	Recurrence: 24 mo AWD:41 mo
Case 3	60	Ic	11,233	YST + HGSC (Ovarian)	TAH + BSO + PLN + omentectomy	TP (6 cycles)	AWD:99 mo

AFP a-Fetoprotein, EC endometrioid carcinoma, CCC clear cell carcinomas, YST yolk sac tumor, HGSC high-grade serous adenocarcinoma, TAH total abdominal hysterectomy, BSO bilateral salpingo-oophorectomy, PLN pelvic lymphadenectomy, PALN para-aortic lymphadenectomy, TP paclitaxel and carboplatin; AWD alive with disease

## Case presentation

### Case 1

A 36-year-old female presented with abdominal distension for over half of a month. Ultrasonography showed a large pelvic mass measuring 2.5*2.5*2.4 cm. A work-up showed α-fetoprotein (AFP) > 1000 ng/ml and cancer antigen 125 (CA125) 28.8 U/mL. Abdominal enhanced CT revealed an intrauterine-cervical canal mass, presence of multiple nodules and greater omentum thickening in the pelvis and fluid in the left fallopian tube.

**Fig. 1:**
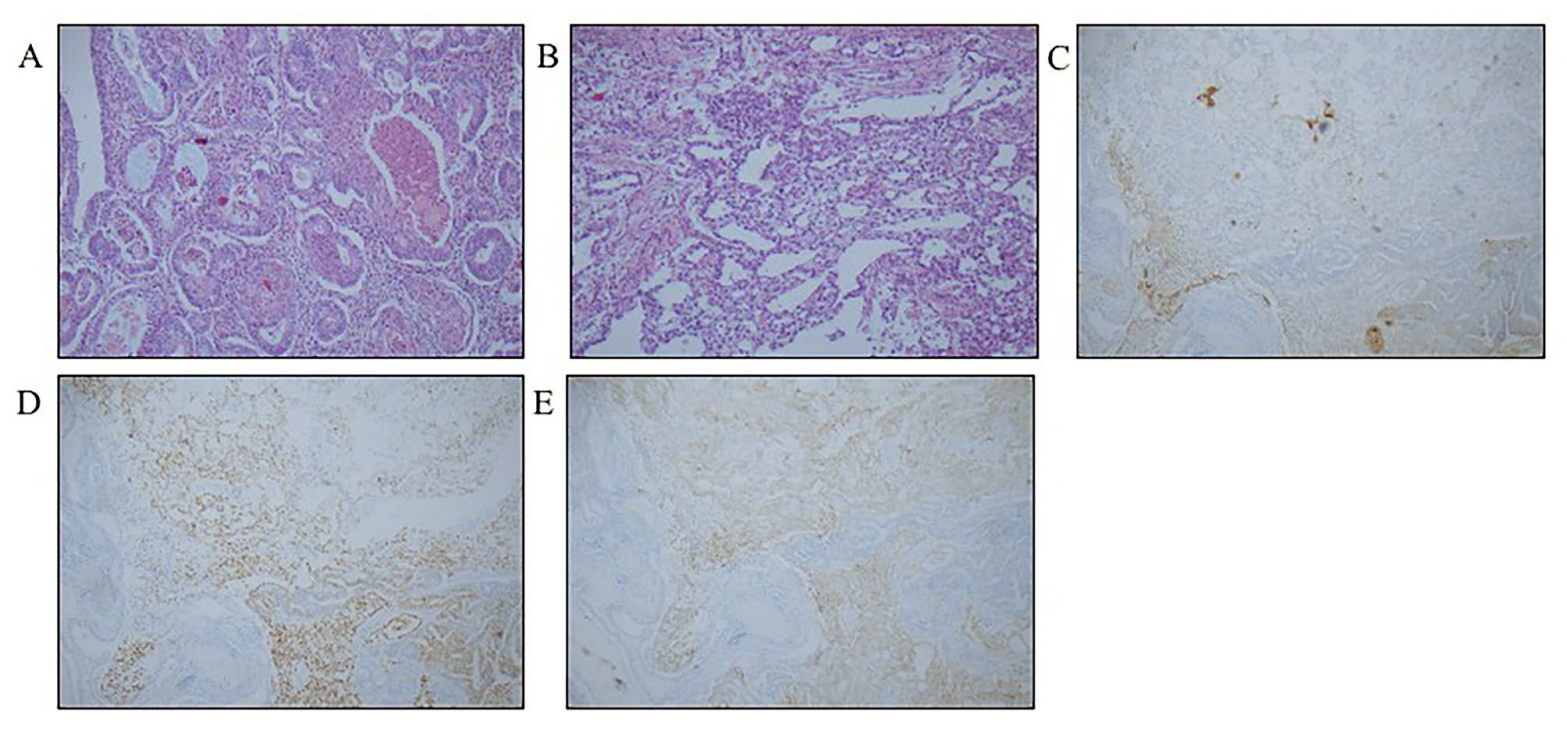
Yolk sac tumour associated with endometrioid carcinoma in case 1. **A**) glandular structures of the yolk sac tumour and endometrioid carcinoma (HE, × 100); **B**) solid growth pattern of the tumour in the yolk sac (HE, × 100); **C-E**) tumour cells of endometrioid carcinoma positive for AFP, SALL4 and GLP3 (SP, × 100)

We performed laparotomy with total abdominal hysterectomy (TAH), bilateral salpingo-oophorectomy (BSO), pelvic lymphadenectomy (PLN), para-aortic lymphadenectomy (PALN) and tumour debulking. The immunohistochemical markers were positive for AFP, Glypican-3 (GLP3), SALL-like protein 4 (SALL4), PMS2, MSH6, MLH1, MSH2, P16, oestrogen receptor (ER), progesterone receptor (PR), Ki-67, P53, NapsinA, cytokeratin (CK), EMA, and CDX2 (caudal type homeobox 2) and were negative for octamer 4 (OCT-4) and CD30, which is consistent with endometrioid carcinoma and clear cell carcinoma with a yolk sac component (G2) (Fig. 1). The final FIGO stage was IVb.

Adjuvant chemotherapy with the TP (paclitaxel and carboplatin) regimen was prescribed for 3 cycles. The AFP level was 107.1 ng/mL after the 3rd cycle of chemotherapy. However, positron emission tomography-computed tomography (PET-CT) revealed intrahepatic metastatic lesions. Therefore, we suggested performing liver ablation and changing the chemotherapy regimen. Unfortunately, she had progressive disease and died 20 months later.

### Case 2

A 55-year-old postmenopausal woman was admitted for vaginal bleeding and abdominal distension. A CT scan demonstrated a cystic solid mass in the bilateral adnexal region with minor ascites. The preoperative AFP was 1190 ng/mL and CA125 was 272.4 U/mL, while carcinoembryonic antigen (CEA) and CA199 were within the normal range. TAH, BSO, PLN, PALN and omentectomy were performed.

**Fig. 2:**
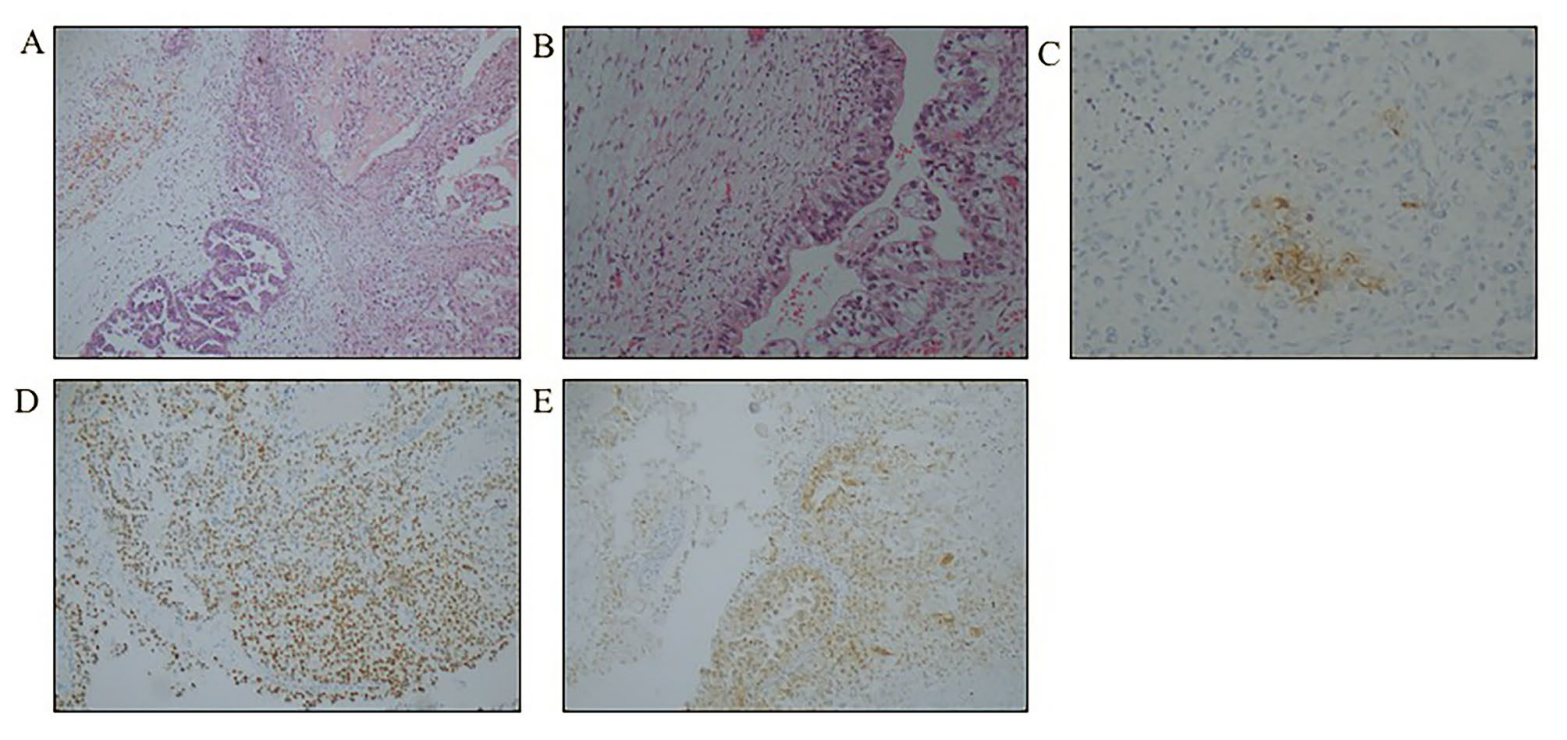
Yolk sac tumour associated with the ovarian tumour in case 2. **(A)** High-grade papillary serous carcinoma mixed with a YST. (HE, × 100); **(B)** Solid growth pattern of the tumour in the yolk sac differentiation area. (HE, × 100); **C-E**) AFP (SP, × 200), SALL4 and GLP3 immunohistochemical staining was positive in the YST component (SP, × 100)

Pathology revealed high-grade serous carcinoma arising from the right ovary with a YST component. Her FIGO stage was IIa. Immunohistochemical staining showed that the germ cells were positive for AFP, GLP3, SALL4, CK, CK7 and EMA and were negative for OCT-4, Napsin A, P16, PR and ER (Fig. 2).

After surgery, she received 6 courses of chemotherapy with the TP regimen. The serum AFP levels were normal after the first chemotherapy cycle. At 2 years postoperatively, she had a recurrent vaginal stump. Therefore, the patient received Niraparib maintenance treatment following reoperation and chemotherapy. At the time of the latest available follow-up, the patient remained clinically and radiologically free of disease.

### Case 3

A 60-year-old woman presented with a complaint of abdominal distension. Before visiting our hospital, she was found to have masses in the left adnexa and right middle abdomen, with massive effusion in the abdominal cavity and pelvic cavity. A recent ultrasound examination showed cystic solid masses measuring 18.1*13.2*10.5 cm and 7.4*5.4*5.4 cm, with obvious blood flow signal in the upper right side of the uterus and left the attachment area, respectively. The tumour marker results were as follows: serum AFP 11233.0 ng/mL, CA125 773.2 U/mL and CA153 39.1 U/ml. The patient underwent exploratory laparotomy. There was 5500 mL of yellow fluid in the abdominal cavity, and the right ovary was replaced by a tumour measuring 20*15*15 cm in size. Most of the tumour was removed, and the fast-frozen pathology result was a malignant tumour. TAH, BSO, PLN and omentectomy were performed.

**Fig. 3:**
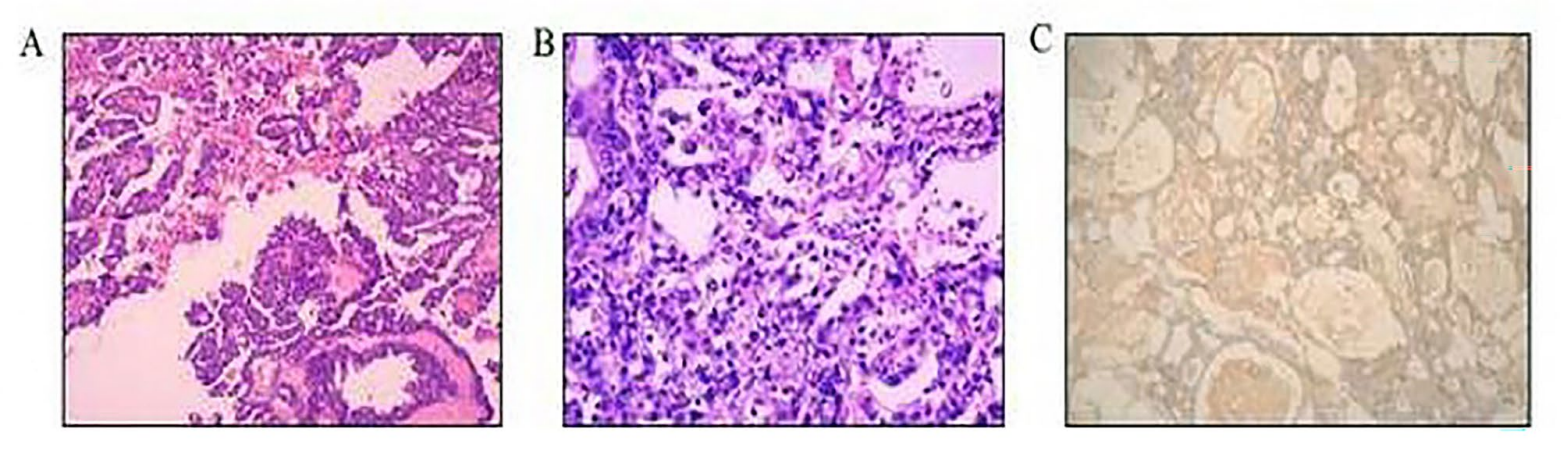
Histologic findings of the ovarian tumour in case 3. **(A)** High-grade epithelial carcinoma (serous and clear cell carcinoma) with the YST (HE, ×100); **(B)** solid area of the YST (HE, ×200); **(C)** tumour cells of the ovarian tumours were positive for AFP (SP, ×100)

Pathologic examination demonstrated an AFP-producing ovarian germ cell tumour (YST) with high-grade epithelial carcinoma (serous and clear cell carcinoma), FIGO stage Ic. Immunohistochemical staining was performed to confirm the histological diagnosis, and the tumour cells were positive for AFP, GLP3, SALL4, CK, CK7, EMA and Ki-67 but negative for OCT-4, NapsinA, P16, ER and PR (Fig. 3).

The patient was treated postoperatively with six cycles of chemotherapy consisting of TP. The serum levels of these markers decreased to normal values after four cycles of chemotherapy. At the current follow-up, there were no signs of tumour recurrence.

## Discussion

YST is a malignant germ cell tumor characterized by endodermal differentiation and usually occurs in young patients [1, 2]. YST mixed with ovarian epithelial tumors or endometrioid adenocarcinoma is rare [5]. YSTs coexisting with endometrial cancer or malignant ovarian tumors are aggressive, and the outcome remains poor. The prognosis is related to the stage of disease and elevated tumor markers [6]. The majority of YSTs mixed with epithelial ovarian carcinoma, especially in postmenopausal patients, appear to be associated with an unfavorable outcome. Endometrioid carcinoma is the most common reported precursor lesion and is usually associated with an endometriotic cyst. Most patients die within 8 months of diagnosis, and only a few are disease-free more than 2 years after diagnosis [6]. In our cases, the patient with endometrioid carcinoma had progressive disease and died 20 months later, and the two other patients were still alive at the last follow-up, suggesting that patients with advanced malignant tumors may have a poorer prognosis than patients with early-stage disease.

Pathologic analyses have revealed that YSTs can be pure or mixed with other germ-cell or epithelial tumors, including embryonal carcinoma, clear cell carcinoma, adenocarcinoma, and serous adenocarcinoma [7–10]. In our cases, the 2 females with YSTs both presented with coexisting epithelial ovarian tumors, and 1 woman presented with endometrioid adenocarcinoma. Some studies have reported that the coexistence of a YST and ovarian malignant epithelial tumor may lead to worse biological behavior [11]. However, it is very difficult to distinguish the YST component from other tumor components. Immunohistochemistry is useful to confirm YST differentiation. GLP3 and SALL4 may be useful markers in the identification of the YST components histologically. GLP3 is an oncofetal protein expressed in fetal liver and malignant tumors of hepatic lineage [12]. SALL4, which originates in somatic tumors, may be useful in identifying YSTs and in the differential diagnosis of ovarian clear cell carcinoma [13]. OCT-4 is positive in dysgerminoma and embryonal carcinoma but negative in YST [14]. In our case, the YST component displayed a characteristic immunophenotype, including positivity for SALL4 and GLP3 and the absence of OCT-4 expression.

AFP is synthesized mainly by the liver and yolk sac and is a useful diagnostic tumor marker for hepatocellular carcinomas [15]. However, plasma AFP in tumors correlates with the YST component [5, 16]. Our report included three patients with AFP-producing gynecological malignant tumors: a 36-year-old female with endometrioid carcinoma and clear cell carcinoma (FIGO IVb), a 55-year-old postmenopausal woman with grade serous carcinoma (FIGO IIa), and a 60-year-old postmenopausal woman with high-grade epithelial carcinoma (FIGO Ic). Previous reports have shown that AFP is important in the clinical diagnosis of YST and in monitoring disease activity and chemotherapy response [3, 17]. The serum AFP levels of the 3 patients were elevated but decreased significantly after surgery.

Because YSTs associated with gynecological malignant tumors are extremely rare, there are no guidelines for treatment. At present, a combination of comprehensive surgery and platinum-based adjuvant chemotherapy is the main means of treatment. Previous studies revealed that YST components mixed with other tumors may be less responsive to chemotherapy [18]. Currently, the gold standard treatment for endometrial carcinoma is surgery: TH with BSO, peritoneal cytology, and lymph node dissection [19]. However, this strategy is not ideal if the patient wishes to become pregnant in the future. The NCCN guidelines include inclusion criteria considering fertility-sparing options for the management of type I, estrogen-dependent endometrioid-type endometrial cancer (EEC): grade (G)1 EEC identified on dilatation and curettage (D&C) confirmed by expert pathology review; the disease should be limited to the endometrium (stage Ia) on MRI (preferred) or transvaginal ultrasound [20]. Several studies have shown that young women with the stage Ia, G1, low-grade endometrioid subtype and nonmetastatic involvement or other risk factors may require conservative management to maintain fertility [21–24]. Markers of poor prognosis, metastasis, and early recurrency may be used to deny fertility-sparing treatment (FST). PTEN and POLE alterations are good prognostic factors of early-stage endometrial cancer; MSI, CTNNB1, and K-RAS alterations are fair prognostic factors, but are associated with a higher risk of recurrence; and PIK3CA, HER2, ARID1A, P53, L1CAM, and FGFR2 are poor prognostic factors [25]. Generally, malignant gynecological tumors with YST components have poor prognosis, especially in advanced patients. Thus, FST is not recommended. In our cases, patients with ovarian YST with FIGO stage Ic high-grade epithelial carcinoma lacked signs of recurrence for 5 years, revealing a potential strategy for patients with early-stage disease who want to preserve fertility. As such, FST may be considered in premenopausal patients with early-stage and low-grade disease with plans to conceive, and individual treatment can be performed according to specific patient conditions after surgery. Further large series and randomized clinical trials are needed to confirm these ideas. In addition, whole-exome sequencing may provide novel insights into the genetic features of these rare biological tumor types.

For inoperable patients with advanced-stage ovarian cancer, neo-adjuvant chemotherapy is a safe and effective alternative. The TP regimen for every 3 weeks remains the gold standard therapeutic regimen [26]. Moreover, Poly (ADP-ribose) polymerase inhibitors (PARP inhibitors) have recently been outraised as maintenance therapy for patients with platinum-sensitive, relapsed ovarian cancer and BRCA half mutation. An III trial demonstrated that patients with platinum-sensitive, relapsed ovarian cancer median progression-free survival was significantly longer with olaparib than with placebo (19.1 vs. 5.5 months), with the estimated hazard ratio being 0.30 (95% CI: 0.22–0.41) [27]. In case 2, the patient with YSTs and ovarian malignant tumor were suffered from adjuvant chemotherapy with the TP and PARP inhibitors maintenance treatment and still alive. Therefore, maintenance PARP inhibitors treatment could be taken into consideration when patients with YSTs and ovarian malignant tumor and BRCA mutation after chemotherapy. However, the use of PARP inhibitors in the neoadjuvant setting has not been studied.

On the other hand, preoperative frailty assessment is fundamental to predict adverse outcomes and to offer them a personalized therapeutic strategy in gynecologic oncology [28, 29]. Modified Frailty Index (mFI) is the most used tool to assess the frailty state of gynecologic oncologic patients [29]. In our cases, mFI of 3 patients were all 0 which did not correlate with post-operative complications and the outcome in terms of OS. The small samples may lead to the results. Further multicentric studies should be conducted to better investigate the role of patients’ frailty in gynecologic oncology.

## Conclusion

We reported three cases of YSTs associated with malignant gynaecological tumours in females. Because mixed YST-carcinoma is a rare malignant neoplasm with high mortality, complete surgical staging combined with chemotherapy may improve the survival rate. In the future, further studies are needed to explore new diagnostic and effective management strategies for YSTs associated with malignant gynaecological tumours.

## Data Availability

The datasets used during the current study available from the corresponding author on reasonable requests.
